# Supplementary material for: Curcumae Radix Decreases Neurodegenerative Markers through Glycolysis Decrease and TCA Cycle Activation
Source: Nutrients. 2022 Apr 11;14(8):1587. doi: 10.3390/nu14081587 (PMC9024545; doi:10.3390/nu14081587)
Supplement: Supplementary file 1 [file nutrients-14-01587-s001.zip › nutrients-1641609-supplementary.pdf]

Supplementary Material

1. Supplementary Figures

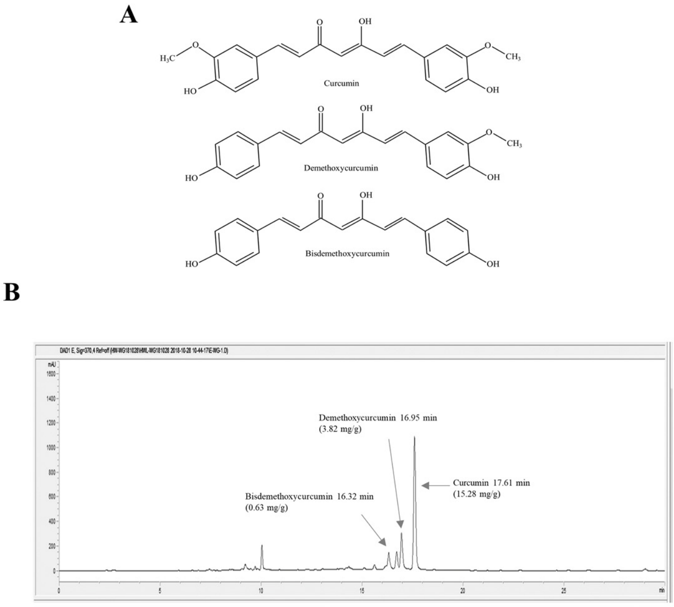

Figure S1. Quatitative Analysis of major ingredients in Curcumae Radix Extract. (**A**) The structures of three reference standards (curcumin, demethoxycurcumin, and bisdemethoxycurcumin), (**B**) Curcumae radix 70% ethanol extraction.

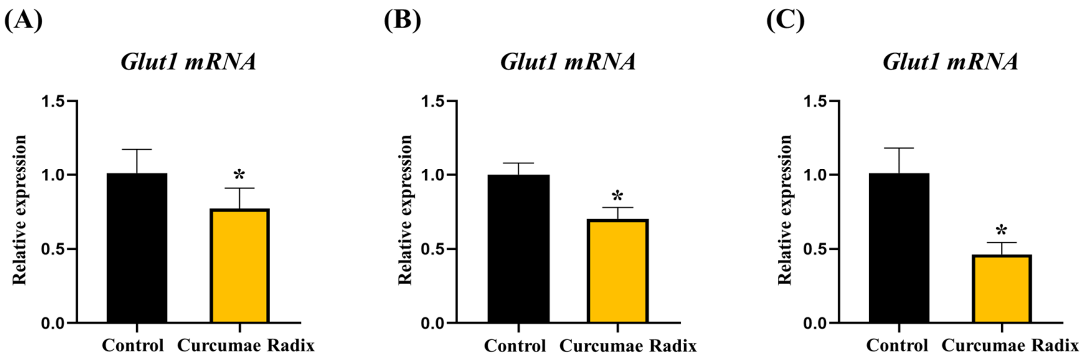

Figure S2. Curcumae Radix reduce glucose transport. (**A**) Glut1 mRNA levels were measured by quantitative RT-PCR in the cerebrum of Curcumae radix group treated CRE and vehicle group treated water (50 mg/kg body weight, n = 6). (**B**) Glut1 mRNA levels were measured by quantitative RT-PCR in DBT cell of Curcumae radix treated. Curcumae radix group treated CRE 4ug/ml. (**C**) Glut1 mRNA levels were measured by quantitative RT-PCR in the cerebrum of Curcumae radix treated Tau transgenic male mice. Curcumae radix group treated CRE and vehicle group treated water (50 mg/kg body weight, n = 3). RPLPO was used for an internal control. The values stand for means +/- S.D. * $p<0.05$ was compared to groups indicated. All experiments were repeated at least 3.

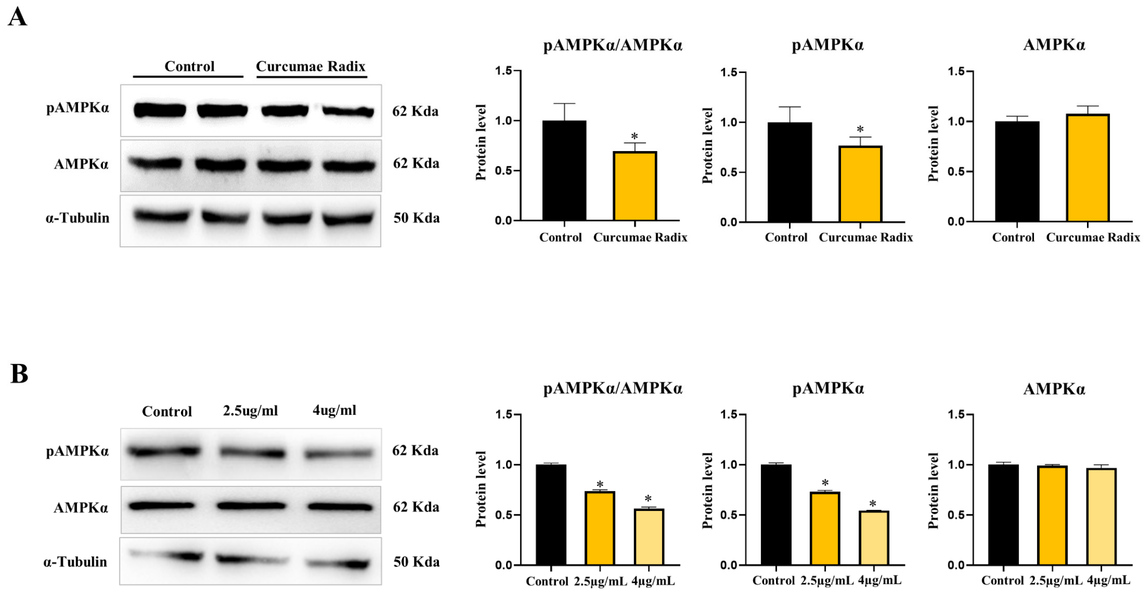

Figure S3. Curcumae Radix improves the energy state. (**A**) Western blot analysis and quantification of Western blot analysis and quantification of phospho-AMPKα (pAMPKα) and AMPKα genes were evaluated in the cerebrum of Curcumae Radix treated male mice. Alpha-Tubulin was used for an internal control. (**B**) Western blot analysis and quantification of Western blot analysis and quantification of pAMPKα and AMPKα genes were evaluated in DBT cell of Curcumae Radix treated. Alpha-Tubulin was used for an internal control. Curcumae Radix group treated Curcumae Radix extract 2.5 ug/ml,4 ug/ml. The values stand for means +/- S.D. * *p*<0.05 was compared to the groups indicated. All experiments were repeated at least 3.
